# Higher Urinary Levels of 8-Hydroxy-2′-deoxyguanosine Are Associated with a Worse RANKL/OPG Ratio in Postmenopausal Women with Osteopenia

**DOI:** 10.1155/2016/6038798

**Published:** 2015-11-09

**Authors:** Carlo Cervellati, Arianna Romani, Eleonora Cremonini, Carlo M. Bergamini, Enrica Fila, Monica Squerzanti, Pantaleo Greco, Leo Massari, Gloria Bonaccorsi

**Affiliations:** ^1^Department of Biomedical and Specialist Surgical Sciences, Section of Medical Biochemistry, Molecular Biology and Genetics, University of Ferrara, Via Borsari 46, 44121 Ferrara, Italy; ^2^Department of Nutrition, University of California, One Shields Avenue, Davis, CA 95616, USA; ^3^Department of Morphology, Surgery and Experimental Medicine, Menopause and Osteoporosis Centre, University of Ferrara, Via Boschetto 29, 44124 Ferrara, Italy; ^4^Department of Morphology, Surgery and Experimental Medicine, Section of Obstetrics and Gynecology, University of Ferrara, Via Aldo Moro 8, Cona, 44124 Ferrara, Italy; ^5^Department of Morphology, Surgery and Experimental Medicine, Section of Orthopaedic Clinic, University of Ferrara, Via Aldo Moro 8, Cona, 44124 Ferrara, Italy

## Abstract

Postmenopausal osteoporosis (PO) is a major public health issue which affects a large fraction of elderly women. Emerging *in vitro* evidence suggests a central role of oxidative stress (OxS) in postmenopausal osteoporosis (PO) development. Contrariwise, the human studies on this topic are still scarce and inconclusive. In the attempt to address this issue, we sought to determine if OxS, as assessed by 8-hydroxy-2-deoxyguanosine (8-OHdG), may influence the level of receptor activator of nuclear factor-*κ*b ligand (RANKL)/osteoprotegerin (OPG) ratio (a central regulator of bone metabolism) in a sample (*n* = 124), including postmenopausal women with osteoporosis, osteopenia and normal bone mass density (BMD). The most striking result that emerged in our study was the independent and positive (beta = 0.449, *p* = 0.004, and *R* ^2^ = 0.185) association between the OxS marker and RANKL/OPG ratio which was found in osteopenic but not in the other 2 sample groups. If confirmed by longitudinal studies, our findings would suggest that OxS is implicated in the derangement of bone homeostasis which precedes PO development. In line with these considerations, antioxidant treatment of postmenopausal women with moderately low BMD might contribute to preventing PO and related complications.

## 1. Introduction

Postmenopausal osteoporosis (PO) is a disease characterized by gradual thickening of bone which leads to a reduced bone mass and an increased risk of fragility fractures [1]. PO occurs mostly because of the decline of oestrogens (especially 17*β*-estradiol, E2) levels produced by cessation of ovarian sex steroid secretion [2]. This endocrine change has major effects on bone remodelling, leading to derangement of the balance between resorption and formation activities of osteoclasts and osteoblasts, respectively [3]. A vast body of evidence suggests that the effects of E2 on bone are mediated by the mutual interaction of receptor activator of nuclear factor-*κ*b (RANK), its ligand (RANKL), and osteoprotegerin (OPG) [4–6].

RANKL exists in both soluble and membrane-bound forms and is expressed by many cell types in bone and bone marrow, including osteoblasts, osteocytes, and activated lymphocytes [7]. Both forms of this protein promote, although with different effectiveness, bone resorption by binding to RANK localized in both precursors and mature osteoclasts, inducing their formation and activation [8]. Osteoblasts are also one of the main sources of OPG which, acting as a decoy receptor that competes with RANKL for RANK, is able to inhibit osteoclastic proliferation and differentiation. The key-role of RANK/RANKL/OPG axis in the pathogenesis of PO has been largely confirmed in preclinical as well as clinical studies which showed that an increase in RANKL-to-OPG ratio can stimulate excessive bone resorption, whereas its decrease can favor bone neoformation [5, 8, 9].

Given the centrality of RANK/RANKL/OPG system in bone metabolism, the systemic factors able to regulate the concentration of these cytokines have acquired great scientific and clinical relevance in recent years. Besides E2 (and calciotropic hormones), there are also a series of inflammatory interleukins (e.g., IL-1 and IL-6) that can alter both RANKL and OPG secretion and activity [5, 6]. Notably, it is now well recognized that the events characterized by burst of these interleukins (i.e., inflammation), but also by physiological decline of E2 (i.e., menopause), are associated with systemic oxidative stress (OxS) [10–12]. This condition can potentially cause the damage against all types of biological molecules and is widely believed to be deeply implicated in the onset and progression of aging-related diseases, including PO [13–16]. More specifically, OxS seems to be a prodromic feature of PO, as suggested by several lines of evidence showing that E2-withdrawal might weaken bone defense against injury induced by reactive oxygen species (ROS) [15, 17, 18]. ROS are, indeed, generated in activated osteoclasts via nicotinamide adenine dinucleotide phosphate oxidase (NOX) and are thought to actively contribute to bone homeostasis “short-circuit” leading to osteoporotic damage [19].

Given these considerations, it was tempting to hypothesize that OxS could play a role in the modulation of RANKL/RANK/OPG triad. On these bases, the aim of the present population-based study was to investigate the potential association between systemic OxS, as assessed by a reliable marker of oxidative damage (urinary 8-hydroxy-2′-deoxyguanosine, 8-OHdG), and serum level of RANKL/OPG ratio in a population sample including healthy, osteopenic, and osteoporotic postmenopausal women.

## 2. Materials and Methods

### 2.1. Subjects

The subjects examined in this study were enrolled among women undergoing bone densitometry evaluation at the Menopause and Osteoporosis Centre of University of Ferrara (Ferrara, Italy), as described elsewhere [20]. The present population-based study was conducted in accordance with the Declaration of Helsinki (World Medical Association, http://www.wma.net) and it was approved by the human research ethics committee of the university. The women were included in the study sample if they were in postmenopausal status, defined as amenorrhea for at least 1 year [21].

Exclusion criteria were use of exogenous sexual hormones (including vaginal estrogens), supplementation with nutritional antioxidants (such as vitamins E, C, and A, beta-carotene, and selenium), vegetarian and vegan diet, chronic diseases (such as diabetes, malabsorption, and cardiovascular disease), or being not diagnosed with a chronic disease, but taking medications (antiobesity medications, thyroid hormones, diuretics, antihypertensive, anticholesterol drugs, etc.).

One hundred twenty-four subjects were found to be eligible and were enrolled in the study after signing an informed consent. Body weight, height and waist circumference were assessed in each enrolled subjects by trained personnel.

### 2.2. Biochemical Assays

Fresh blood samples were obtained from antecubital vein from all subjects between 8.30 and 10.00 am, after fasting for at least 8 h. After 30 minutes of incubation at room temperature (RT), blood samples were centrifuged (3000 g for 10 min), and the obtained serum was stored at −80°C until analysis. Commercially available Enzyme-Linked Immunosorbent Assays (ELISAs) kits were performed, according to the manufacturer's instructions.

Serum level of total (free plus bound) soluble RANKL was assayed by Human sRANKL (total) ELISA (catalog number RD193004200R, purchased from BioVendor Research and Diagnostic Products, Modrice, Czech Republic). In brief, standards, quality controls, or samples (100 *μ*L each) were incubated in microplate wells precoated with monoclonal anti-human sRANKL antibody. After a 16–20-hour incubation (at 2–8°C), the plate was washed and incubated for 60 minutes at RT with biotin labelled polyclonal anti-human sRANKL antibody. After a further washing step, streptavidin-HRP conjugate was added and incubated for 60 minutes (RT). Then the plate was rewashed and the remaining conjugate was allowed to react with the substrate solution containing hydrogen peroxide and tetramethylbenzidine (TMB). After stopping the reaction, the plate was read at 450 nm. The concentration of RANKL in the serum samples was estimated from the standard curve and expressed as pmol/L (detection limit: 0.4 pmol/L). The intra-assay CV was 9.3%, whereas the interassay CV was 11.0%.

Serum concentration of OPG was detected by OPG ELISA kit (catalog number EK0480, Boster Biological Technology Co., Ltd., China). One hundred *μ*L of either standards or properly diluted serum samples was added into anti-human OPG antibody precoated wells. After incubation (90 minutes at 37°C), biotinylated anti-human OPG antibody was added into each well and the plate was reincubated at the same temperature for a shorter time interval (60 minutes). Following the first washing step, a solution containing avidin, biotin, and peroxidase was added into each well of the plate which was then incubated for further 30 minutes (37°C) and thoroughly washed one more time. Afterwards, TMB color developing agent was added and, eventually, the absorbance was read at 450 nm. The serum concentration of OPG was estimated from the standard curve and expressed as pmol/L (detection limit: 1 pmol/L). The intra-assay CV was 5.3%, whereas the interassay CV was 7.0%.

Serum concentration of bone-specific alkaline phosphatase (BAP) was detected by OCTEIA Ostase BAP immunoenzymometric assay (catalog number AC-20F1, purchased by Immunodiagnostic Systems Ltd., Boldon, UK). Fifty *μ*L of either standards, controls, or serum specimens was pipetted into streptavidin precoated wells and subsequently mixed with a biotin-labelled BAP-specific monoclonal antibody. After incubation (1 hour at RT), substrate reagent solution (i.e., p-nitrophenyl phosphate) was added into each well. A further incubation (15 minutes at RT) was followed by the addition of stop solution into each well. The absorbance was finally read at 405 nm (subtracting blank reading at 650 nm). The serum concentration of BAP was estimated from the standard curve and expressed as *μ*g/L (detection limit: 0.7 *μ*g/L). The intra-assay CV was 4.1%, whereas the interassay CV was 5.5%.

Serum concentration of C-terminal telopeptides of Type I (CTX-1) was measured by serum Cross-Laps ELISA kit (catalog number AC-02F1, purchased by Immunodiagnostic Systems Ltd., Boldon, UK). Briefly, 50 *μ*L of either standards, control, or serum samples was pipetted into streptavidin precoated wells followed by the addition of the antibody solution (containing biotinylated monoclonal murine antibody plus monoclonal murine antibody conjugated with peroxidase). After 2 hours of incubation at RT, wells were washed and then chromogenic substrate (TMB) solution was added. Measurement of the absorbance at 450 nm with 650 nm as reference was made within two hours after the addition of the stop solution. The concentration of CTX-1 in the serum samples was obtained by standard curve and was expressed as ng/mL (detection limit: 0.020 ng/mL). The intra-assay CV was 2.2%, whereas the interassay CV was 7.7%.

High sensitivity C-reactive protein (Hs-CRP) serum concentration was assessed by commercial kit Hs-CRP the EiAsyTM Way  (catalog number CAN-CRP-4360, purchased from Diagnostics Biochem Canada Inc., Dorchester, CAN). Twenty *μ*L of either each calibrator, control, or properly diluted serum sample was pipetted into mouse anti-CRP precoated wells. After 30 minutes of incubation (RT) followed by a washing step, anti-CRP monoclonal conjugated with horseradish peroxidase was added into each well. After 15 minutes at RT, followed by a washing step, TMB substrate solution was added and, eventually, the absorbance was read at 450 nm within 20 minutes after the addition of the stop solution. The concentration of Hs-CRP in the serum samples was estimated from the standard curve and expressed as ng/mL (detection limit: 10 ng/mL). The intra-assay CV was 9.5%, whereas the interassay CV was 9%.

Urine concentration of 8-OHdG was detected by competitive 8-OHdG EIA kit (catalog number SKT-120-96, purchased from StressMarq Biosciences Inc., Victoria, BC, Canada). Fifty *μ*L of either standard or properly diluted urine specimens was added into wells precoated with goat anti-mouse IgG. Afterwards, 2 equal volume aliquots of 8-OHdG-acetylcholinesterase conjugate and 8-OHdG monoclonal antibody were added to each well. After an overnight incubation (4°C) Ellman's reagent [5,5′-dithiobis-(2-nitrobenzoic acid)] was added to each well. The absorbance was finally read at 405 nm. The concentration of 8-OHdG in the urine samples was obtained by standard curve and was expressed as ng/mL (detection limit: 0.033 ng/mL). The intra-assay CV was 7.8%, whereas the interassay CV was 6.4%. The 8-OHdG concentration was normalized to urinary creatinine concentration and expressed as ng/mg creatinine.

Urinary creatinine determination was performed by a picric acid method [22]. Briefly, 50 *μ*L of either standard or properly dilute urine samples was added into microplate well and mixed with 200 *μ*L of a working solution containing 25 mM picric acid (purchased form Sigma-Aldrich, St. Louis, MO, USA) and 130 mM NaOH. The 490 nm absorbance was read after 30 minutes of incubation at RT and the obtained concentration was expressed as mg/dL (detection limit: 0.1 mg/dL). The intra-assay CV was 5.3%, whereas the interassay CV was 7.6%.

All the above ELISAs were assayed by a Tecan infinite (M200 Tecan Group Ltd., Männedorf, Switzerland) microplate spectrophotometer.

### 2.3. Bone Densitometry Assessment

Areal bone density was assessed at lumbar spine, hip, and total body by Discovery dual energy X-ray absorptiometry scanner (Hologic Inc., Bedford, MA). PO was diagnosed when BMD *T*-score (the number of standard deviations below the average for a young adult at peak bone density) was lower than 2.5 standard deviations from BMD peak at either femoral neck or lumbar spine, according to WHO guidelines [23]. In accordance with these criteria, women with *T*-score at either skeleton area between −2.5 and −1.0 were classified as osteopenic and those with a value higher than −1.0 as normal.

### 2.4. Statistical Analysis

SPSS 18.0 for Windows (IBM, Chicago, IL, USA) was used for statistical analysis. All variables were first analyzed for the normal distribution by the Kolmogorov-Smirnov and the Shapiro-Wilkinson test. Differences between groups were checked by one way analysis of variance (ANOVA) and Kruskal-Wallis for normally and non-normally distributed variables, respectively. Univariate analysis (by Pearson's or Spearman's test, depending on the distribution of the variable) was performed to check the associations between selected variables. Simple and multiple linear regression analysis were performed using base-10 logarithm transformed values of RANKL, OPG, RANKL/OPG, and 8-OHdG. We used log-transformed variables for these analyses to meet the assumption of normality of regression residuals. A two-tailed probability value <0.05 was considered statistically significant.

## 3. Results

The main characteristics of the 124 postmenopausal women enrolled in the present study are shown in Table 1. Osteoporotic and osteopenic women were older (*p* = 0.009) and presented lower BMI (*p* = 0.04) and waist circumference (*p* = 0.03) compared to those with BMD values within normal range. In accordance with the diagnostic criteria, total hip, neck, and lumbar spine BMD, as well as the correspondent *T*-score values, were significantly (*p* < 0.01) higher in controls with respect to osteopenic and osteoporotic women. In contrast, serum level of Hs-CRP, RANKL, OPG, RANKL/OPG ratio, CTX-1, BAP, and urinary level of 8-OHdG did not significantly vary among the three sample groups.

The possible association of 8-OHdG with the other biochemical markers and BMD values was initially checked by simple correlation analysis (Table 2). From this test it emerged that the DNA damage marker was significantly correlated only with RANKL (*p* = 0.003) and RANKL/OPG ratio (*p* = 0.002).

**Figure 1:**
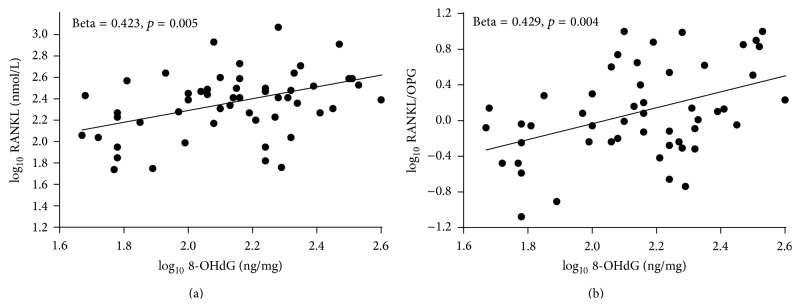
Box plots of the correlations: log_10_ 8-OHdG versus log_10_ RANKL (right); log_10_ 8-OHdG versus log_10_ RANKL/OPG.

Afterwards, we checked the association between 8-OHdG and the two cytokines within each sample group (Table 3). As displayed in the table and in Figure 1, the OxS marker resulted to be significantly and positively correlated with RANKL (*p* = 0.005) and RANK/OPG (*p* = 0.004) merely in the osteopenic group, with a percentage of variance explained equal to 18.0 and 18.2%, respectively. Of note, the linear standardized coefficient for the association between 8-OHdG and OPG was negative (beta = −0.196) and, although not statistically significant (*p* = 0.098), markedly higher than those obtained among controls (beta = −0.037) and osteoporotic (beta = −0.089) women.

Finally, in order to unveil if the correlations found to be significant in the osteopenic group were independent of potential confounding factors, we performed two multiple regression models including age, years since menopause, BMI, waist circumference, and Hs-CRP as covariates (Table 4). These analyses showed that both associations, 8-OHdG versus RANKL and 8-OHdG versus RANKL/OPG, retained their significance even after multiple adjustments.

## 4. Discussion

In the present study, we evaluated the possible link between PO and OxS, as assessed by 8-OHdG, by various angles. The analyses of the data obtained revealed that this OxS marker was not significantly associated with diagnosis of osteopenia/osteoporosis; BMD of different skeleton area; resorption/formation bone markers. In contrast, we found that higher level of 8-OHdG was strongly and independently related to increased serum concentration of RANKL and RANKL/OPG among postmenopausal women with osteopenia but not among those with normal BMD or osteoporosis.

In line with our results, several previous works [15, 16, 24, 25] did not find any significant difference in the peripheral level of OxS between osteoporotic and healthy postmenopausal women. In contrast, some of these studies [15, 16], along with others [14, 26, 27], found an inverse, although statistically weak, correlation between OxS and femoral neck and/or lumbar spine BMD. In our point of view, the reasons of these discrepancies mostly lay in the different indicators employed for peripheral OxS determination. Indeed, almost all the aforementioned studies dealt with markers (such as malondialdehyde, hydroperoxides, and F2-isoprostanes) which are all derived by lipid peroxidation. This cascade reaction is markedly different from the DNA repair process yielding 8-OHdG. Differently from the former, lipoperoxidation leads to the formation of several by-products, which are very reactive and can markedly amplify the initial ROS-induced oxidative spark. Furthermore, DNA oxidative injury merely occurs inside the cells, whereas the targets of lipoperoxidation are disseminated both in and out of the cytosol (e.g., membranes of cell and organelles and lipid moiety of circulating lipoproteins). Consistent with these observations, the precious systematic work by Kadiiska and colleagues suggests that there could be different forms of OxS, and each might bring about the rise of a different series of peripheral markers [28].

Our finding of the absence of a detectable increase in systemic OxS in relation to PO occurrence does not rule out that reactive species can play a role in the development of this bone disease. The gathered results also showed, indeed, that OxS might be an effective influencing factor of RANK/RANKL/OPG triad, which plays a paramount role in the pathogenesis of PO and other metabolic bone diseases [5, 6, 29, 30]. This discovery adds to the current literature, because, to the best of our knowledge, it is the first time that such interaction is found in human subjects. Conversely, there is abundant supportive evidence from* in vitro* experiments on various cell lineages such as mouse osteoblasts, human MG63, and primary bone marrow cell cultures [14, 31]. More in detail, Baek and coworkers showed that oxygen peroxide can promote the number and activity of osteoclasts and RANKL expression, but not OPG. Of interest, these effects were abolished upon adding catalase, a potent H_2_O_2_-scavenger [14]. Increase in endogen ROS burden leading to enhancement of RANKL production of osteoclast precursor cells can be derived by NOX activation or by a reduced expression of nuclear factor (erythroid-derived 2-) like 2 (Nrf2), as shown in a recent work on Nrf-2 knockout mice [13]. Noteworthily, the partial activation of this redox-sensitive transcription factor, which regulates the expression of several genes encoding essential antioxidant enzymes, resulted in inhibition of osteoclast differentiation [13].

The clinical importance of the present study derives from the fact that RANK/RANKL/OPG axis is now widely regarded as one of the most promising molecular targets for novel therapeutic approaches in the management of bone diseases [4, 30]. Accordingly, the inhibition of RANKL by denosumab (a fully human antibody against RANKL) was more effective at reducing the occurrence of vertebral fractures than the traditional drugs [9]. In spite of these encouraging outcomes, there is still an intense demand for alternative, nonpharmaceutical (and, at least hopefully, safer) interventions on this high-incidence disease. In this context the* in vitro* and animal data highlighting the protective effects on bone elicited by various antioxidants such as lycopene [32], resveratrol [33], and tocotrienol [34] are promising.

Unfortunately, the human observational studies set out to examine the effects of antioxidants on bone health are still sparse and controversial [35] and do not allow translating the preclinical evidence in an effective antiosteoporotic treatment. Moreover, the interpretation of the epidemiological is difficult because most of these studies are affected by important limitations such as cross-sectional design [24, 36, 37] and lack of measurement of circulatory antioxidants concentration which should accompany the evaluation of nutrients intake by dedicated questionnaires [24, 38]. The latter point is of primary importance, because the bioavailability of these compounds depends on the food matrix consumed and on genetic variability and physiological condition of the subjects [35]. Besides, the findings may be biased by the interference of other nutrients as suggested by the authors of one of the few longitudinal studies on this field [38]. Indeed, examining a sample of 891 women, Macdonald et al. observed significant negative correlations between BMD and nutrients, in particular vitamin E (from diet alone) and polyunsaturated fatty acids (PUFAs). The researchers suggest that the strong association that was also found between PUFAs and vitamin E intake (*r* = 0.822, *p* < 0.001) could account for this unexpected result and, as consequence, this vitamin could simply represent a surrogate marker for fat intake [38]. Disappointing data were also obtained in a cross-sectional study by Wolf et al. [36], where dietary and total intake, or serum concentration of vitamin E, *β*-carotene, lycopene, and other antioxidants, failed to be associated with BMD in women (*n* = 11068, aged 50–79 years). Contrariwise, a clear beneficial effect of *α*-tocopherol was found in a recent longitudinal study which showed that low intake and serum concentration of the vitamin were both associated with an increased rate of bone fracture in both elderly women (*n* = 61422) and men (*n* = 1138) [39]. Finally, similar bone protective effects of carotenoids [40] or vitamin C [41] emerged from data collected in Framingham Osteoporosis Study.

Overall, the published epidemiological studies, although presenting some controversies and design issues, appear to support the commonly held belief that antioxidant-rich fruits improve bone health and are strongly suggestive of a beneficial role of these bioactive molecules [31]. However, one must be aware that it is not yet completely clear if these osteoprotective effects are merely exerted by an antioxidant pathway or by the simple restoring of mineral balance and/or vitamin K bioavailability [35, 36].

Thus, well-designed, randomized, controlled studies are warranted to confirm the findings from the animal studies on bone loss and subsequent development of osteoporosis. In our view, however, the concept that has to be borne in mind is the following: PO is a multifactor and multifaceted disease, and, thus, OxS should not be considered as the unique enemy to defeat. Owing to these considerations, it is conceivable to assume that antioxidants alone could not represent the definitive treatment for PO but more likely as therapeutic adjuvant of well-established antiosteoporotic drugs. The indication that emerged from our investigation was that this type of supplements might benefit postmenopausal women with osteopenia. Indeed only in those subjects, where bone remodeling cycle is altered, but not still completely compromised, the RANK/RANK/OPG system appeared to be sensitive to the elevation of 8-OHdG. Therefore, OxS, during this condition defined as prelude of PO, could contribute to uncoupling the balance between bone resorption and formation, thus guiding the process of bone degeneration to the “definitive” osteoporotic damage. Arresting this process, before BMD is not too low (*T*-score < 2.5), is very important to prevent from onset of PO and related fragility fractures [31].

Finally, some important limitations of the study must be acknowledged. First, the design of the study was cross-sectional, thereby precluding our ability to establish any temporal relationship between the markers examined. Therefore, longitudinal investigations are mandatory to draw a definitive appreciation of causal nature of OxS with respect to alteration in circulatory level of RANKL and RANKL/OPG. Second, the lack of a full nutritional assessment of the sample subjects makes it difficult to rule out the fact that dietary antioxidant intake might interfere with the assessed level of 8-OHdG and, hence, with the reliability of study outcomes. However, to attenuate the influence of this factor, all the individuals reporting the use of antioxidant supplements or to follow a vegetarian diet were excluded a priori from the study. Third, the serum level of RANKL and OPG might not reflect the levels and activity of these cytokines in bone microenvironment, and a portion of them could originate from nonskeletal sources, in particular inflammation [4, 5]. In this regard, in the attempt to limit the potential interference of this factor on our statistical outcomes, we included Hs-CRP in the multivariate analysis. Finally, a limitation of the fact that RANKL/OPG is not a suitable marker for PO diagnosis could appear. On the contrary, it may represent a potential strength of the mounting consensus around the use of this peripheral index for monitoring bone health and antiosteoporotic therapy response of patients with bone diseases [42].

We would like to also underline some other strengths of the present work. To the best of our knowledge, this is the first study that provides* in vivo* data in support of the interaction between RANKL/OPG and OxS among women with high risk of osteoporosis. Noteworthily this correlation resulted to be independent by potential confounders such as age and measures of body fat. Moreover, we consider as further study strength the use of a widely recognized reliable marker of DNA oxidative damage such as urinary 8-OHdG [28, 43].

## 5. Conclusion

In conclusion, our findings demonstrate the existence of a positive association between systemic OxS and serum level RANKL/OPG ratio in osteopenic but not in normal and osteoporotic postmenopausal women. Thus, the data obtained, although warranted confirmation by longitudinal studies, suggest that women with moderately low BMD could be the target population for antioxidant-based interventions aimed at preventing osteoporosis-related bone loss and fracture.

## Figures and Tables

**Table 1:** Principal characteristics of normal, osteopenic, and osteoporotic postmenopausal women.

	Normal BMD	Osteopenia	Osteoporosis	Statistics
	(*n* = 25)	(*n* = 59)	(*n* = 40)	*p*
Age, yr	54.0 ± 4.2	56.2 ± 4.5	57.7 ± 4.7	0.01^*∗*^
Years since menopause, yr	3 (1–9)	5 (2–10)	7 (4–12)	0.09^#^
BMI, kg/m^2^	24.2 (23.0–27.4)	23.1 (21.5–27.3)	23.2 (22.1–26.7)	0.16^#^
Waist circumference, cm	85.9 ± 10.9	83.2 ± 8.4	82.5 ± 8.9	0.05^*∗*^
DXA parameters				
L. spine BMD, g/cm^2^	1.10 ± 0.91	0.92 ± 0.11	0.83 ± 0.09	<0.01^*∗*^
L. spine *T*-score	0.1 (−0.6 to 0.3)	−1.8 (−2.1 to −1.2)	−2.5 (−2.8 to −1.8)	<0.01^#^
F. neck BMD, g/cm^2^	0.81 (0.79–0.86)	0.67 (0.63–0.71)	0.62 (0.57–0.64)	<0.01^#^
F. neck *T*-score	−0.3 (−0.6 to 0.0)	−1.6 (−2.0 to −1.2)	−2.1 (−2.5 to −1.8)	<0.01^#^
Total hip BMD, g/cm^2^	0.93 ± 0.08	0.81 ± 0.07	0.74 ± 0.07	<0.01^*∗*^
Total hip *T*-score	−0.1 (−0.4 to 0.4)	−1.1 (−1.5 to −0.8)	−1.5 (−2.0 to −1.2)	<0.01^#^
Biochemical markers				
Hs-CRP, mg/L	1.5 (0.6–3.7)	1.1 (0.6–2.1)	1.2 (0.4–2.6)	0.54
CTX-1, ng/mL	0.47 ± 0.21	0.46 ± 0.39	0.52 ± 0.30	0.47^*∗*^
BAP, *μ*g/L	30.3 ± 1.3	31.0 ± 1.0	25.1 ± 1.3	0.24^*∗*^
RANKL, pmol/L	270 (201–362)	255 (157–347)	281 (166–365)	0.18^#^
OPG, pmol/L	8.1 (7.3–10.6)	11.0 (6.9–16.6)	12.0 (5.7–18.6)	0.78^#^
RANKL/OPG	31.0 (18.2–64.1)	20.1 (11.5–60.1)	21.2 (11.4–34.2)	0.31^#^
8-OHdG, ng/mg creatinine	143 (109–189)	154 (109–208)	159 (112–212)	0.35^#^

Data presented are expressed as mean ± standard deviation for normally distributed variables; median (interquartile range) for not normally distributed variables.

^*∗*^ *p* value by Kruskal-Wallis; ^#^ *p* value by ANOVA.

BMI: body mass index; BMD: bone mass density; L.: lumbar; F.: femoral; Hs-CRP: high reactivity C-reactive protein; CTX-1: C-terminal telopeptide of type I collagen; BAP: bone-specific alkaline phosphatase; OPG: osteoprotegerin; RANKL: receptor activator of nuclear factor kappa-B ligand; 8-OHdG: 8-hydroxy-2′-deoxyguanosine.

**Table 2:** Simple correlation between 8-OHdG and RANKL, OPG and RANKL/OPG, BMD values, and bone resorption/formation markers (total sample, *n* = 124).

	8-OHdG
L. spine BMD	−0.13
F. neck BMD	0.04
Total hip BMD	0.03
CTX-1	0.07
BAP	0.06
RANKL	0.263^*∗*^
OPG	−0.116
RANKL/OPG	0.277^*∗*^

^*∗*^ *p* < 0.01 by Pearson's analysis of base-10 logarithm transformed values of the 2 variables.

BMI: body mass index; BMD: bone mass density; L.: lumbar; F.: femoral; CTX-1: C-terminal telopeptide of type I collagen; BAP: bone-specific alkaline phosphatase; OPG: osteoprotegerin; RANKL: receptor activator of nuclear factor kappa-B ligand; 8-OHdG: 8-hydroxy-2′-deoxyguanosine.

**Table 3:** Simple linear regression analysis for the relationship between urinary level of 8-OHdG and serum levels of RANKL, OPG, and RANKL/OPG ratio in normal, osteopenic, and osteoporotic postmenopausal women.

		Normal BMD	Osteopenia	Osteoporosis
RANKL	*B* (DS)	0.325 (0.389)	0.554 (0.164)	0.018 (0.212)
	Beta	0.188	0.423^*∗*^	0.014
	*R* ^2^	0.035	0.180	0.001

OPG	*B* (DS)	−0.048 (0.288)	−0.256 (0.175)	−0.093 (0.256)
	Beta	−0.037	−0.196	−0.089
	*R* ^2^	0.001	0.038	0.004

RANKL/OPG	*B* (DS)	0.368 (0.549)	0.879 (0.262)	0.161 (315)
	Beta	0.160	0.429^*∗*^	0.086
	*R* ^2^	0.026	0.184	0.007

^*∗*^ *p* < 0.001.

Beta: standardized regression coefficient; *B*: nonstandardized regression coefficient; BMD: bone mass density; OPG: osteoprotegerin; RANKL: receptor activator of nuclear factor kappa-B ligand; 8-OHdG: 8-hydroxy-2′-deoxyguanosine.

**Table 4:** Multiple regression analysis for the relationship between 8-OHdG and RANKL and RANKL/OPG, among osteopenic postmenopausal women (*n* = 59).

Predictors	Dependent variable		Dependent variable	
	RANKL		RANKL/OPG	
	Beta	*p* value	Beta	*p* value
8-OHdG	0.469	0.002	0.449	0.004
Age	0.071	0.694	0.119	0.644
Years since menopause	0.031	0.849	0.015	0.930
BMI	0.016	0.939	0.059	0.274
Waist circumference	0.001	0.998	−0.049	0.812
Hs-CRP	−0.115	0.411	−0.079	0.523

	*R* ^2^ = 0.202		*R* ^2^ = 0.185	

Beta: standardized regression coefficient; BMI: body mass index; BMD: bone mass density; Hs-CRP: high reactivity C-reactive protein; RANKL: receptor activator of nuclear factor kappa-B ligand; 8-OHdG: 8-hydroxy-2′-deoxyguanosine.
